# Is Scarlatina a Bovine Malady?

**Published:** 1893-01

**Authors:** 


### SELECTIONS.

### IS SCARLATINA A BOVINE MALADY?

By Professor McCall.

*Abstract from the Opening Address at Veterinary College, Glasgow.*

The subject I have selected to discuss on this occasion is the grave malady in mankind known by the name of "Scarlet Fever." It is unusual for veterinary surgeons to choose a human disease as the theme of their remarks upon such occasions as the present, because it is a field of research in which they cannot be said to be in at home.

Speaking for myself, I can assure you I have no desire to constitute myself an authority upon any of the many diseases which affect the human body, and my sole reason for taking up the subject is, that it has been publicly proclaimed in various papers, professional and non-professional, that the scarlet fever of man originates in the cow, and by the milk secretion of the affected animal is conveyed to man. In proof so far of this, I shall now read to you a copy of a paragraph which appeared in *The Bailie* of date 17th of August last: "The rather sharp epidemic of scarlet fever on the south side has been nipped in the bud, thanks to our system of the notification of disease. On the first day day 103 cases were reported at the Sanitary Office, on the second 105, on the third 100, and on the fourth fifteen The worst cases were removed to Belvidere, and not only was the city dairy *found*, but the farm and cows in Renfrewshire which caused the trouble. The cows were examined by Drs. Russell and Munro, and found *to be suffering from scarlatina.* With regard to human diseases and their close relation to that of animals, there is more in it than is yet dreamt of in medical philosophy." And by way of keeping up the attention of the public, a reference is again made to it in the next weekly issue in the following terms: "The Health Committee of the Town Council have decided to ask the assistance of the experts of the Local Government Board to make experiments with the matter got from the pustular eruption on the teats of the cow, *said to have caused* the recent outbreak of scarlet fever on the south side."

Such are the statements made, and I have anxiously scanned the pages of *The Bailie* for the report of the experts of the Local Government Board; but as yet all is silent, and as no one has contradicted the statement "*that the cows were suffering from scarlatina*," I consider it to be a duty incumbent on me to calmly review the circumstances and conditions which have seemingly warranted such a serious charge being brought against the poor cows.

It has been, as you see, alleged that the human malady, scarlatina, can originate in the cow, and by ingestion of the milk of the affected animal the bovine scarlatina can, in its turn, become the human scarlatina. Need I say that such an allegation, in the face of the daily large consumption of the milk of the cow by the human subject, is one of grave importance. In the twenty-second annual report on the operations of the Sanitary Department of the city of Glasgow for the year 1891, I observe that 3,115 individuals were attacked by this disease, and from another source I am informed that from 17,000 to 18,000 individuals die in England annually from scarlatina. If, then, the malady originates in the cow, and is imported into the body of mankind by partaking of the milk as an article of diet, the question, I think, may fairly be asked, Is it wise and an actual necessity for the human subject to run such a risk? Would it not be in the interests of the human family to discard the use of milk?

We are told that by using the milk of tuberculous cows, the hitherto healthy human subject may become the victim of consumption, and such an inference, in my opinion, founded on experiments, is justifiable; but notwithstanding all this, I am not aware that any medical authority has alleged that tuberculosis originates in the cow, and descends from her to the human family.

We are also too familiar with the fact that typhoid fever has been produced in the human subject by the ingestion of milk; but no medical authority has proclaimed that typhoid fever originates in the cow, although, strange to say, it was the tracing and connecting of an outbreak of enteric fever in Islington by Dr. Ballard in 1870, with the milk supplied to the affected persons, which first connected the cow with human maladies, and has since laid her open to grave suspicion.

In 1878 there was a serious outbreak of diphtheria in Kilburn and St. John's Wood, and as it was alleged that "it was *possible to assert* that none of the agencies by which milk, after being taken from the cow, may become infective, could with any shadow of probability have been in operation," the fiat went forth that the disease, diphtheria, originated in the cow, and is transmissible in the milk of the affected cows to the human subject.

The allegation having now been widely accepted as an established fact, the cow having become the accredited originator of human diphtheria, what may not her possibilities be? And, accordingly, we find in 1885 she has added another laurel to her crown, and been declared the originator of the disease, human scarlatina.

[Professor McCall here gave a careful *résumé* of the evidence in the "Hendon case."]

Let us now more particularly turn our attention to the outbreak referred to in *The Bailie;* and as it is facts we are in quest of, and not theories or opinions entertained by medical and veterinary experts, I shall allow parties to speak for themselves, and, accordingly, I shall first call upon the proprietor of the inculpated cattle to speak for himself.

Thomas Wilson :—" I am tenant of the farms of Jaapston and Aboon-the-Brae, and entered into possession of them in November 1891. I reside along with my wife, three children, and servants at Jaapston, and my father, mother, and their family and servants reside at Aboon-the-Brae. We milk twenty-eight cows at Jaapston, and twenty-five at Aboon-the-Brae, but the cattle and servants are all kept distinct, and the one farm steading is fully one mile from the other. All the cows on Aboon-the-Brae were on Jaapston first for a time, but on 29th May last they were sent up and kept there.

" On or about 11th July I observed that two or three cows on Aboon-the-Brae had sore teats, and the trouble gradually spread through them, but the cows on Jaapston had no eruptions on their teats until 25th of July. The milk was taken as usual once a day to Glasgow, and there delivered by my milk-van into the premises of the Coöperative Dairy Company. The milk of the cows at both farms was used daily by our families and servants, and until the 4th of August we ourselves were all in perfect health, and as regards the cattle, although they had sore teats and were troublesome to milk, they fed well, milked well, and appeared to me to be in perfect health.

"On 4th August my eldest child (a boy of two years) took unwell, and our doctor saw him the same evening. He said that he feared it would prove a case of scarlet fever, and in consequence, the child and his mother and a baby in her arms were at once placed in rooms at Jaapston away from us all. My wife alone attended to the affected child, and they had their meals by themselves. The child was only poorly for a few days, and although shut up in the room with his mother, was never confined to bed.

"On 7th of August the manager of the Dairy Company came out to my farm and reported that cases of scarlet fever had cropped up among persons receiving milk from the Company, and that the first case had occurred on the 4th August. This was the first intimation I had had casting suspicion on our milk, and we ourselves at both farms were regularly using the milk, and we have since, without intermission, continued to use it. I told him of my child also being attacked with scarlet fever on 4th August.

"As, save the eruptions on the teats, the cattle at Aboon-the-Brae were in health, and as there was no scarlet fever among the individuals there, it was suggested and arranged to send the Jaapston cattle up to Aboon-the-Brae, and this was done betwixt four and five o'clock on the morning of the 8th August. On the same day the milk was returned by the Dairy Company, and the same day Dr. Monro, medical officer for Renfrewshire, paid us a visit and examined all the persons on Jaapston and Aboon-the-Brae. He found one girl (Jennie Deans) at Jaapston with a sore throat. and she said that she felt it sore the previous day. She continued to work at Jaapston after the cows left, and was better in two days. This girl was one of the three milkers of the Jaapston cows until they were taken up to Aboon-the-Brae; but she was kept at Jaapston, and the other two girls went up with the cattle.

"On 9th August, Dr. Monro, Dr. Russell, medical officer, Glasgow, and Professor McCall came to Aboon-the-Brae and inspected the whole of the cows. By this time almost all the cows that were giving milk either had eruptions on their teats or were recovering from the effects of these—about half the number had recovered.

"On 12th August Professor McCall returned and again examined the cows at Aboon-the-Brae, and the same day he picked out *twenty-two* cows which he said had recovered from the eruption. These *twenty-two* cows were, by his advice, washed with disinfectants, and it was arranged that the milk which they gave should be sent into Glasgow next morning.

"The remaining *thirty-one* cows which had sore teats were, on the advice of Professor McCall, taken back to Jaapston for treatment, and that the milk which they gave might be made into cheese or churned, which was done, and no complaint was ever made against the butter.

"On 13th August the milk of the *twenty-two* cows on Aboon-the-Brae was sent into Glasgow, but the Dairy Company refused to take delivery, and it was brought back. We now commenced to make butter at Aboon-the-Brae as well as at Jaapston.

"On 23d August, by arrangement, the milk was again sent into Glasgow and received by the company, and it was continued to be sent and received for one week; but on 30th August a telegram was sent me to discontinue sending it, as two fresh cases of scarlatina had been reported. We now arranged to sell all the milk as 'lapper' for churning and making butter, and there has been no complaint.

"Regarding the disease affecting my cows, I don't think it has any connection with scarlet fever in man. I have seen the same kind of eruption many a time, but always in cows that were being milked. It spreads from one cow to another by the hand in milking. It is what we call the 'pock.' and if you have a sore on your hand or a cut you will get the 'pock' from the cow. Here is the proof. I blistered my thumb when using the hay-rake, and on milking I got this 'pock.' It was very painful for some days, especially at the arm-pits. Several of the milkers have had a 'pock,' but we attended to them and they were not bad. About the beginning of August, one of the milkers (Maggie Pollock) had a sore hand, and two pocks formed betwixt the fingers, and as she could not milk we had no particular need for her, so we allowed her to go home on the 6th August and she returned on the 20th all right. It was alleged this woman had scarlet fever, but there was no truth in it.

"As formerly stated, the mother and two of our children—the oldest and youngest—were shut up in a room, and as my other child had been in their company, I thought it safest that he should be removed to his aunt's at Neilston, and he was removed on 4th August, the day his brother took unwell. He remained with his aunt till the 24th August; but in consequence of his cousin—a girl of eleven years—being attacked with scarlet fever, he was removed to my sister's house. She resides in Holehouse, Neilston, but the child had only been there two days when one of my sister's children contracted scarlet fever, and on being informed of this, late on the evening of the 26th, I decided to have the child brought home to Jaapston. On 28th August, at night, he complained of a sore throat, and next morning the doctor saw him, and as he had a strong suspicion that the trouble was also scarlet fever, he was now placed along with his brother in his mother's apartments, and attended to by her. His attack of scarlet fever was a mild one, and he, as well as his brother, made a good recovery. The village of Neilston was very bad with mild cases of scarlet fever *shortly before my boy* went down, and consequently before my other child at Jaapston had been attacked.

"Regarding how my first child got scarlet fever, I do not know, unless from some of the poor children which come out from Glasgow and reside for a fortnight at Sundayhill and Waterside. These children frequently pass the farm of Jaapston, as the road is a public one past it, and my front doors open on the roadside. The children frequently loiter about, looking at what is going on, and they occasionally purchase a halfpenny worth of milk and drink it.

"During the whole period, myself and all the farms have drunk the milk of the cows and used it without the least restriction, and not one of us has been unwell, and even the two boys affected with scarlet fever have drunk it daily and continue to do so; and some of the cows' teats are still sore. Indeed, at Jaapston we had no other milk to drink than the milk of the cows with eruptions on their teats, as the cows with sound teats, or teats at all events nearly so, were up at Aboon-the-Brae."

Such is Mr. Wilson's voluntary evidence, and given without the least hesitancy or reservation ; and now we will take up Mrs. Wilson's evidence.

"I am the wife of Thomas Wilson, and reside at Jaapston. On the evening of the 4th August one of my children became affected with scarlet fever, and he and his baby brother and myself were ordered by the doctor to reside in rooms by ourselves. I had assisted in milking the cows, and when I was shut up with my children I had a pock on one of my fingers, and in a day or two four other pocks formed on the same hand. The hand and arm were swollen and very painful up in the arm-pit, and I experienced pain and difficulty in bathing and attending to my baby. Unfortunately, somehow or other, I also gave the pock to my infant, and a large pock formed on the upper surface of the big toe some days after we were shut up. It was painful, and the child fretted, but that was all. I never had the scarlet fever, and neither the baby nor myself took the infection from the boys, nor did my husband or any of the servants. I was informed that our milk had given scarlet fever to persons in Glasgow, and was vexed to hear it; but as I was certain that our milk got the infection after it left Jaapston, I never discontinued using it, and as Professor McCall had sent down to Jaapston all the cows with sore teats, and kept the cows with sound teats at Aboon-the-Brae, I had no other milk to use; and my children, including those affected with scarlet fever, and the baby and myself, all drank of the milk freely and without any bad effects. The attacks of scarlet fever which my two boys had were very mild."

Such is Mrs. Wilson's evidence, and I will only refer to that of another, viz.: William McMaster.

"I am vanman to Mr. Wilson, and deliver the 'butts' containing the milk to the dairy in Glasgow. I never had scarlet fever, nor any one about the farms, excepting the master's two wee boys. I use the milk daily.

"I am generally in Glasgow at the dairy to deliver the milk at six in the morning. I carry the 'butts' into the dairy and empty them into large cans which are measured. The empty 'butts' I generally get all back with me, but sometimes not. It is out of the large cans filled by me that the milk is taken by the Dairy Company's servants, and thereafter filled into other cans for distribution. At the dairy the milk of both farms is mixed, and I have seen the milk from another farm (which generally arrives shortly after ours) poured into the same cans as contain our milk, provided sufficient had been taken out to receive it. The week before scarlet fever broke out, owing to the Dairy Company having more milk than they could sell, I had difficulty in getting empty butts, and when I got them, some of them had a most horrible smell, and had to be boiled in the boiler at home before we could get rid of the smell. I don't know how we got the scarlet fever, but I was told that *one* of the Dairy Company's shopwomen had had a mild attack of scarlet fever."

From the standard work of Dr. Bristowe, fifth edition, page 162, I read—"The contagion of scarlet fever is very powerful and diffusive. It may be carried considerable distances by the atmosphere, certainly through the whole dimensions of a large ward; it clings to clothes and other fomites with great tenacity, and may lie latent, yet capable of action for an indefinite period; it is *liable to infect milk* and other articles of food exposed to its influence. Scarlet fever occurs only as the result of contagion, usually conveyed by the means which have already been indicated It seems that it may also be transmitted by direct inoculation. For there is reason to believe that it can be imparted by inserting the fluid of the scarlatina vesicles, beneath the cuticle of persons who have not yet had it, and it is certain that women at the time of parturition are specially liable to take it, receiving it then in some cases from the fingers of the accoucheur.

"The incubation of scarlet fever is shorter than that of most diseases of the same class. It usually varies between six and eight days, but is occasionally longer, though very often less. Many cases, indeed, of undoubted authenticity have been recorded in which it certainly did not exceed twenty-four hours."

Now let us analyze the history of this outbreak of cow disease, and the evidence for and against its being the same disease as scarlatina of the human subject. On the 11th of July, in a herd of twenty-five cows located on the farm of Aboon-the-Brae, two or three are discovered with eruptions on their teats, the disease spreads from cow to cow giving milk. In fourteen days later, viz., 25th July, a similar disease commences among the twenty-eight cows on Jaapston, which is a mile distant, and gradually spreads among them.

Regarding this disease, there is no proof of its being infectious, but abundant proof that it is contagious and inoculable. Abundant proof to show that the human hand charged with the virus can inoculate the teat of the cow if there be the slightest scratch upon it, and not only so, but that the operator can inoculate himself or herself.

Now, what is this disease? We are told that it is "bovine scarlatina," a specific disease dependent on the presence of a microbe termed "micrococcus-scarlatina," "streptococcus-scarlatina," and which Dr. Klein *alleges* he had found in the eruptions on the teats, but not in the milk drawn from the affected animals.

It is also alleged that the milk of a cow affected with this disease, if drunk by a human being, produces human scarlatina, but the allegation overshoots the proof. Dr. Klein exonerated the milk, but incriminated the fluid contained in the local eruptions on the outside of the teats. How, then, can it be said that the scarlatina virus originates in the cow?

It might with as much accuracy be said that the hairs of the human head originate scarlet fever, when an outbreak of the disease is traceable to a lock of hair cut from the head of an affected person, and which by the desire of loving friends had been forwarded.

Admitting, however, that the "virus," the "infective agent," is contained in the eruptions on the teats, and *not* in the milk as secreted, and that this organism is under the microscope indistinguishable from that of the alleged organism of human scarlatina, has it been proven that the milk or liquid in the eruptions or the desquamating cuticle produced scarlet fever in those individuals who drank the milk of the affected cows on Jaapston and Aboon-the-Brae? I think not. It is the same disease which commenced on 11th July as existed on 4th August, and during these twenty-four days the milk was drunk by men, women and children employed and connected with both farms, and also by hundreds of individuals within the city of Glasgow, and not one solitary case of scarlet fever resulted.

It may be said that the scarlatina organisms were not sufficiently numerous in the milk to take effect until the bulk of the cows became affected. Even making such a concession, which, I hold is not warranted by all we know of the life history, reproduction, and propagation of organisms which produce specific diseases; still we have several other formidable objections to offer. The virus of scarlatina fever, we are told, resides in the eruptions on the teats; be it so. Mrs. Wilson becomes inoculated in five distinct parts of her body, and the scarlatina virus gives unmistakable evidence of its having taken effect and been absorbed into the lymph circulation, and yet she remains in health, and has no scarlatina eruptions on her body. Not only so, but the same person inoculated with the specific virus freely drinks scarlatina milk (the very milk alleged to give scarlatina to the inhabitants of Glasgow), and, strange to say, she has no scarlatina. Further, this same unhappy person, inoculated at five points with scarlatina virus, and freely ingesting scarlatina milk, gives the disease, said to be scarlatina, to her babe by inoculation; the virus takes effect; she feeds the babe on her own scarlatina milk, and what more milk is required she supplies it from the cows laboring under scarlet fever, and, curious to relate, the babe thrives, and exhibits not one single symptom of scarlatina.

Scarlatina, according to Dr. Bristowe, is an inoculable disease; the mother and the babe are successfully inoculated. Scarlatina, we are told, can be propagated by ingesting (drinking) milk containing the scarlatina virus; the mother and her child drink this milk and are not affected.

Mr. Wilson becomes also inoculated with scarlatina, and drinks daily scarlatina milk for weeks and remains in health.

Jeanie Deans becomes inoculated with bovine scarlatina, the virus of which when drunk gives human scarlatina, and she also drinks the scarlatina milk and escapes.

Maggie Pollock is also subjected to the double crucial test; her body is inoculated with the scarlatina virus, and it takes, and she daily drinks the scarlatina milk which is killing probably some, and affecting many of the inhabitants of Glasgow, and she also escapes.

The whole human population on the farms of Jaapston and Aboon-the-Brae drink freely of scarlatina milk and escape; the milkers of the animals have sores on their hands similar to those on the teats of the cows, but these sores neither in the cow nor man bear the least resemblance to the sores or eruptions of scarlet fever.

We come now to the two little boys who, without doubt, were attacked by, and suffered from, the disease, genuine human scarlatina. The first boy was seized on 4th August, and there and then he is with his mother and her babe immured within the walls of Jaapston. Where did he get the infection? By drinking the scarlatina milk of the cows? It cannot be, because those who were inoculated with the scarlatina virus from the teats, and also drank the milk of the affected cattle, in no case had a scarlatina eruption or indisposition, such as affected this boy. Not only so, but this child for some days almost lived on the milk of the scarlatina cows, and yet made a good recovery. It was, you will remember, considered a possible explanation how it came to be the fact that for twenty-four days the inhabitants of Glasgow drank the milk of the scarlatina cows and suffered not; that the virus was not sufficiently concentrated; but here we have genuine human scarlatina plus bovine scarlatina daily ingested, yet the child makes a good recovery.

On the same day that this boy shows the first symptoms of scarlatina at Jaapston, the first case of scarlatina in Glasgow from persons using the Jaapston milk is also reported. Is there a connection betwixt the two outbreaks? I think not; but, either way you put it, the impression left on my mind as to the origin of the outbreak is the same.

Twenty-four days' drinking of scarlatina milk produces no scarlatina in those drinking it, in one day a solitary case at Jaapston, and 103 cases in Glasgow result, no more outbreaks at Jaapston traceable to the milk, several more outbreaks in Glasgow. I say, put it either way you like, the origin of the fever points to a human and *not* a bovine source. The *milk* has now undoubtedly become a *vehicle of contamination*, and consequently those who now drink it, after it leaves the dairyman's premises, fall victims to scarlet fever.

I am not particular whether you inculpate the boy at Jaapston, or more probably a boy, girl, woman, or man, employed by the Dairy Company in Glasgow; all I maintain is that the evidence points to a human source of contamination.

Coming to the second boy attacked at Jaapston, you remember he was sent away to his aunt's at Neilston the same day as his brother took unwell. In three weeks after entering his aunt's house her daughter is seized with scarlet fever, but it is clear that the boy did not transmit the disease to her. The trouble was in the village before he went and before his elder brother was attacked. He moves to another aunt's house at Holehouse, where scarlet fever also exists, and in two days one of her children is seized. The same evening he is taken home to Jaapston, and in two days the poor little wanderer also falls a prey to the prevailing malady—human scarlatina. Neither this boy nor any other person in the villages of Neilston or Holehouse drank the milk of the scarlatina cows at Jaapston, and no medical officer or any other medical gentleman has called in question the milk supply of these villages, and still human scarlatina prevails; and I feel constrained to say that, if there had been no eruptions on the teats of Mr. Wilson's cows, no one would have incriminated the cows, although scarlatina had followed the path of the milk distribution.

Cows frequently have eruptions on their teats; indeed, it would be difficult to find a herd of milch cows without some of the members of the herd having similar eruptions on their teats.

In the Hendon outbreak a great deal of stress was placed on the fact that some of the cows were desquamating freely, as desquamation of the scurf skin is a prominent symptom in human scarlatina. Singular, so far to relate, in this outbreak of so-called bovine scarlatina, I observed no animal desquamating; but, with the experience I have gained in feeding and milking a large number of dairy cows, I attach little importance to a dairy cow or feeding heifer shedding her hair and throwing off the scurf skin. Keep the animals tied up in a warm cow-shed, and give them warmed boiled food and hot meal drinks, and both hair and cuticle will be shed. The Jaapston cows were lying out and not receiving hot food, and hence they were not desquamating.

In the series of interesting experiments detailed (although not undertaken as experiments), we have found that neither the milk nor lymph contained in the sores on the teats of the cows said to be affected with bovine scarlatina, produced human scarlatina when ingested, nor did human scarlatina result when the individuals were not only inoculated, but also largely fed on so-called bovine scarlatina milk.

Further, it was found that two boys laboring under human scarlatina, fed daily on bovine scarlatina milk, made a good recovery. And now we shall refer briefly to experiments undertaken with the express intention of transmitting human scarlatina to cows.

The experiments to which I will now refer were carried out by Professor M'Fadyean, of the London Veterinary College, and are given in detail in a report presented to the Agricultural Department by Professor Brown "On Eruptive Diseases of the Teats of Cows in Relation to Scarlet Fever in Man."

Experiment I.—"On 24th October, 1887, I inoculated an Ayrshire cow with blood taken from the arm of a scarlatina patient, and on the evening of that day it gave birth to a calf. The same animal had administered to it on the 2d of November about three pints of water which had been used to wash two scarlatina patients in the state of desquamation, and which contained a great quantity of epithelium. On the 7th of December this cow was inoculated with a sub-culture of Klein's streptococcus scarlatina, and obtained from Dr. Klein. The cow was kept under close observation for one month after the inoculation. Result, negative."

Experiment II.—"Calf of No. 1 had an injection of Klein's streptococcus. Result, negative."

Experiment III.—"Cow had a plug of cotton wool soaked in water containing flakes of scarlatina desquamation inserted into the vagina. The cow had given birth to a calf two hours prior. Result, negative. Same cow six weeks later was inoculated with a sub culture of Klein's streptococcus scarlatina. Result, negative."

Experiment IV.—"Calf of cow No. 3 sucked its mother for a month, thereafter had administered a pint of water with scarlatina cuticle. Result, negative."

Experiment V.—"Two calves inoculated with twenty minims each of a sub-culture of Klein's streptococcus scarlatina. Result, negative."

We thus see that, by the usual method adopted for transmitting specific disease, viz. by ingestion and inoculation, singly or combined, the human malady, "scarlatina," has not yet, and in all probability never will be, transmitted to cows.

And now one word as to the duties of medical officers under such circumstances. Undoubtedly, it will be at once admitted that it is the bounden duty of medical officers to take every possible precaution by way of ensuring the health and safety of the public. If, therefore, the medical officers find that outbreaks of scarlatina and other infectious diseases are following in the wake of the distribution of milk from some particular source, it is their duty to have the distribution of milk from that particular source at once stopped, and the cause of contamination thoroughly investigated. I have presented before you a full statement of the whole facts of the Hendon and Jaapston cases by way of showing that no valid evidence has ever been adduced to prove the existence of scarlatina as a bovine disease. On the contrary, I have clearly shown how, for the space of twenty-four days, the people at Jaapston and Aboon-the-Brae were freely drinking the milk of cows alleged to be affected with bovine scarlatina, and yet every one of these people remained in a state of perfect health. At the same time it is perfectly clear that this same milk when sent to Glasgow produced scarlet fever in those who drank it; and this fact clearly shows that this milk must have got contaminated with human scarlatina after being drawn from the cows. Most decidedly it is their bounden duty to instantly put a stop to its distribution. To "cast to the dogs" the milk of fifty-three cows, as in the case referred to, is a serious matter—a heavy pecuniary loss—a loss which no ordinary farmer can contend with; but, notwithstanding, the protection of the lives of the lieges justly demands it. The milk is infected, and it matters not who is to blame; its use as a human beverage cannot be allowed. Is it for the dairy farmer producing the milk, and the dairyman retailing the same, to discover the source of infection, and cut it instantly off? and in accomplishing this I am sure that both parties will have the hearty and able assistance of Local Authorities and their medical and veterinary officers.

At present, however, the whole or almost the whole pecuniary loss falls upon the producer of the milk, the vendor refusing to receive the milk, canceling his contract for the time to run, and entering into a new agreement with another party. This is not fair under any circumstances, and very unfair if the infection has gained access to the milk after leaving the producer's premises. I would therefore suggest that the dairy farmers forwarding milk to Glasgow should create a Benefit Society, appoint one or more medical and veterinary officers and a law-agent to attend to and represent their interests; and I would advise that a Society on similar lines be created by persons engaged in vending milk within the city; and no doubt, betwixt the well-directed efforts of both Societies, outbreaks of human scarlatina and other specific diseases connected with the consumption of milk would be speedily and with certainty traced to their source and stopped, and thus pecuniary loss averted and human life protected and prolonged.—*The Veterinary Record.*

